# A collection of hexactinellids (Porifera) from the deep South Atlantic and North Pacific: new genus, new species and new records

**DOI:** 10.7717/peerj.9431

**Published:** 2020-07-09

**Authors:** Cristiana Castello-Branco, Allen G. Collins, Eduardo Hajdu

**Affiliations:** 1 National Museum of Natural History & National Systematics Laboratory of NOAA Fisheries Service, Smithsonian Institution, Washington DC, USA; 2 Museu Nacional, Universidade Federal do Rio de Janeiro, Rio de Janeiro, Brazil

**Keywords:** *Advhena*, *Bolosoma*, *Euplectella*, *Poliopogon*, Shinkai, NOAA, Manned submersible, ROV

## Abstract

This article describes or redescribes four hexactinellid sponges, namely *Poliopogon amadou, Euplectella sanctipauli* sp. nov., *Bolosoma perezi* sp. nov. and *Advhena magnifica* gen. et sp. nov. *P. amadou, E. sanctipauli* sp. nov. and *B. perezi* sp. nov. represent new findings for the South Atlantic deep-sea fauna, including the first record of *Bolosoma* for this ocean. *Advhena magnifica* gen. et sp. nov., on the other hand, was collected by NOAA oceanographic expeditions in the North Pacific (Pigafetta Guyot).

## Introduction

In deep-sea environments, corals and sponges form ecologically important structures that provide habitats for other organisms (Fuller et al., 2008), and as a result are commonly associated with biodiversity hotspots (Freiwald et al., 2004). Hexactinellids or glass sponges (Porifera, Hexactinellida) are siliceous marine sponges that occur on both hard and soft sediments. These sponges are diverse and abundant in deep-sea environments, frequently being the dominant component in deep benthic communities (200–6000 m depth), where they secrete considerable amounts of silica (Reiswig, 2002). The class Hexactinellida has classically been divided into two subclasses, Amphidiscophora and Hexasterophora, based on microsclere form, amphidiscs or hexasters, respectively. Whereas Amphidiscophora has contained one order only (Amphidiscosida), Hexasterophora has traditionally been divided into four orders (Aulocalycoida, Hexactinosida, Lychniscosida, Lyssacinosida). Parts of this classification have recently been altered to reflect phylogenetic considerations (Dohrmann et al., 2017; Kersken et al., 2018; Dohrmann, 2019). While the subclass designation into two reciprocally monophyletic groups, Amphidiscophora and Hexasterophora, has been retained, present classification divides the latter into orders Lychniscosida, Lyssacinosida, Sceptrulophora and a diverse assemblage of Hexasterophora *incertae sedis* (Reiswig, 2002; Van Soest et al., 2019).

Despite their ecological importance as ecosystem engineers providing framework habitat for many taxa (Barthel, 1992; Krautter et al., 2001; Beazley et al., 2013; Hajdu et al., 2017), hexactinellids most likely have their diversity severely underestimated (Dohrmann, Collins & Wörheide, 2009; Van Soest et al., 2012). The entire South Atlantic has had only 33 species reported to date, the majority (28) from the Southwestern Atlantic (Schulze, 1887, 1904; Burton, 1932, 1940; Uriz, 1988; Lopes, Hajdu & Reiswig, 2005, 2007, 2011; Menshenina et al., 2007; Tabachinick et al., 2009; Hajdu, 2010; Carvalho et al., 2016), and most occurring on the continental slope. To increase knowledge on hexactinellid biodiversity, we describe here new species and new records based on dredging, ROV operations, and manned submersibles conducted in two sectors of international seabeds, the Southwest Atlantic and Northwest Pacific.

## Materials and Methods

**Figure 1:**
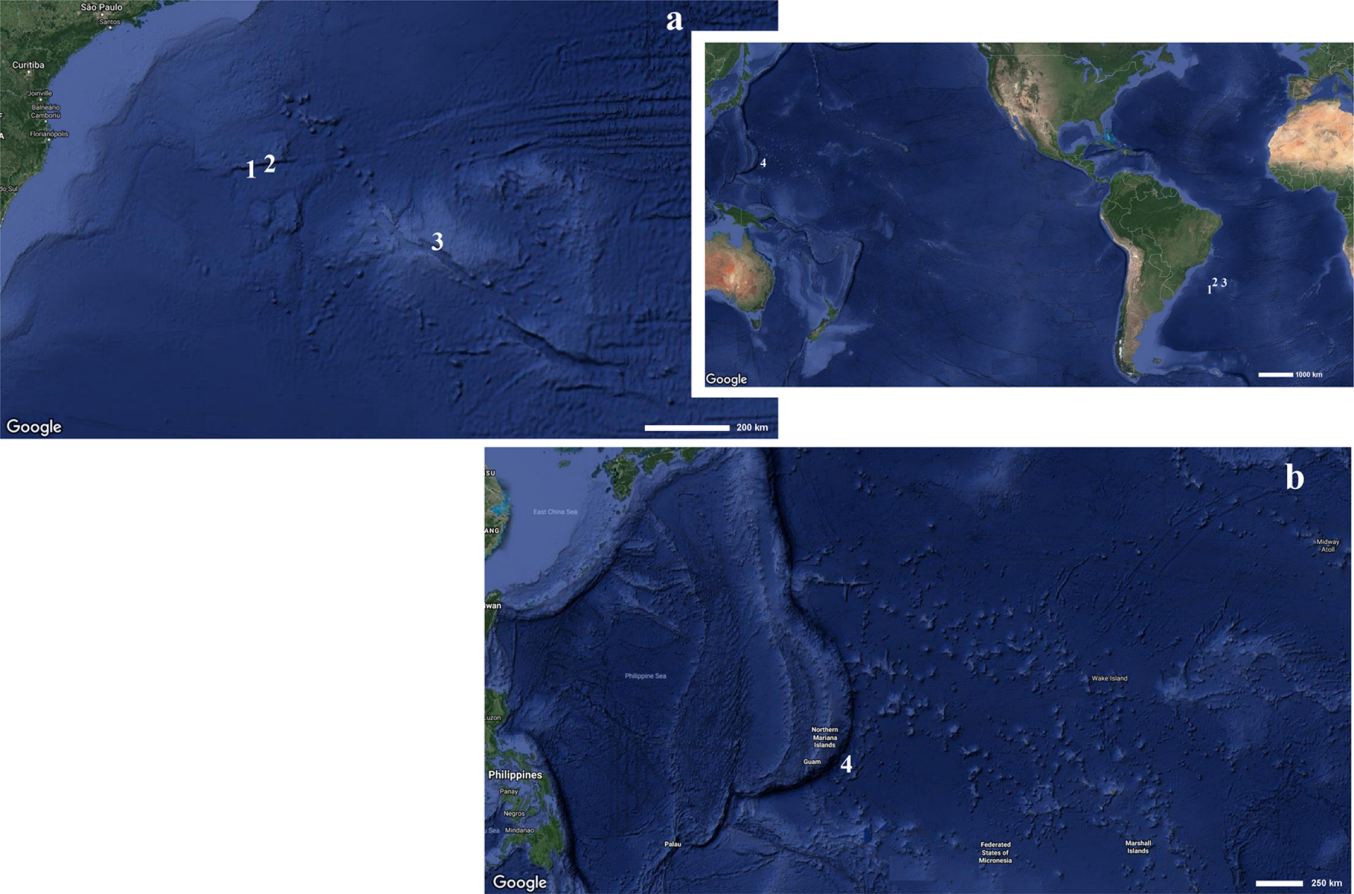
Map indicating the collecting locality of hexactinellid specimens. Details of the (A) Pacific and (B) Atlantic regions. Number (1) *Poliopogon amadou*. Number (2) *Euplectella sanctipauli* sp. nov. Number (3) *B. perezi* sp. nov. Number (4) *Advhena magnifica* sp. nov. (Map data ©2019 Google).

Data presented here came from the PROERG (CPRM; many vessels, dredging), Iata Piúna/QUELLE 2013 (JAMSTEC; S/V “Yokosuka*”*, “Shinkai*”* manned submersible) and Deepwater Exploration of the Marianas (NOAA Ship Okeanos Explorer and “Deep Discoverer” ROV) oceanographic expeditions. The first two to the Rio Grande Rise (aprox. 30°48′0″S/35°36′0″W) and São Paulo Ridge (aprox. 28°11′60″S/41°0′0″W, South Atlantic), in 2011 and 2013; and the last one to the Marianas’ Trench (Pacific Ocean), in 2016 (Fig. 1). Specimens are deposited partly in the Porifera collection of Museu Nacional/UFRJ, and partly in the Smithsonian National Museum of Natural History collection. The material was studied and described following standard procedures outlined by Reiswig & Stone (2013), with the difference that isolated spicules were not captured in nitrocellulose filters, but were pipetted directly onto glass slides or metal stubs. Micrometric data on spicules was obtained from 30 measures for each category, unless indicated otherwise. Abbreviations used: MNRJ—Porifera Collection at Museu Nacional/UFRJ (Rio de Janeiro, Brazil), USNM—Smithsonian National Museum of Natural History (Porifera collection), Programa da Elevação do Rio Grande (PROERG), Companhia de Pesquisa de Recursos Minerais (CPRM), Japan Agency for Marine-Earth Science and Technology (JAMSTEC), Quest for the Limit of Life (QUELLE).

The electronic version of this article in Portable Document Format (PDF) will represent a published work according to the International Commission on Zoological Nomenclature (ICZN), and hence the new names contained in the electronic version are effectively published under that Code from the electronic edition alone. This published work and the nomenclatural acts it contains have been registered in ZooBank, the online registration system for the ICZN. The ZooBank Life Science Identifiers (LSIDs) can be resolved and the associated information viewed through any standard web browser by appending the LSID to the prefix http://zoobank.org/. The LSID for this publication is: urn:lsid:zoobank.org:pub:19DC8DB7-AE38-4CBD-8DBE-F90799979352. The online version of this work is archived and available from the following digital repositories: PeerJ, PubMed Central and CLOCKSS.

## Results

Phylum Porifera *Grant (1836)*

Class Hexactinellida *Schmidt (1870)*

Subclass Amphidiscophora Schulze (1886)

Order Amphidiscosida *Schrammen (1924)*

Family Pheronematidae *Gray (1870)*

Genus *Poliopogon* Thomson (1877)

Diagnosis

Body is fan-like, where the concave side represents the atrial cavity. Basalia are in relatively broad tufts and include some monaxons with clavate distal ends and two-toothed anchors. Choanosomal, hypodermal and hypoatrial spicules are pentactines, rarely stauractines and tauactines. Uncinates usually consist of only one type. Dermalia and atrialia are pinular pentactines and rare hexactines. Microscleres are amphidiscs (from one to three kinds) and combinations of microhexactines and pentactines (in some species also stauractines, diactines, monactines and spheres) (Tabachnick & Menshenina, 2002).

*Poliopogon amadou* Thomson (1877)

(Figs. 2 and 3)

**Figure 2:**
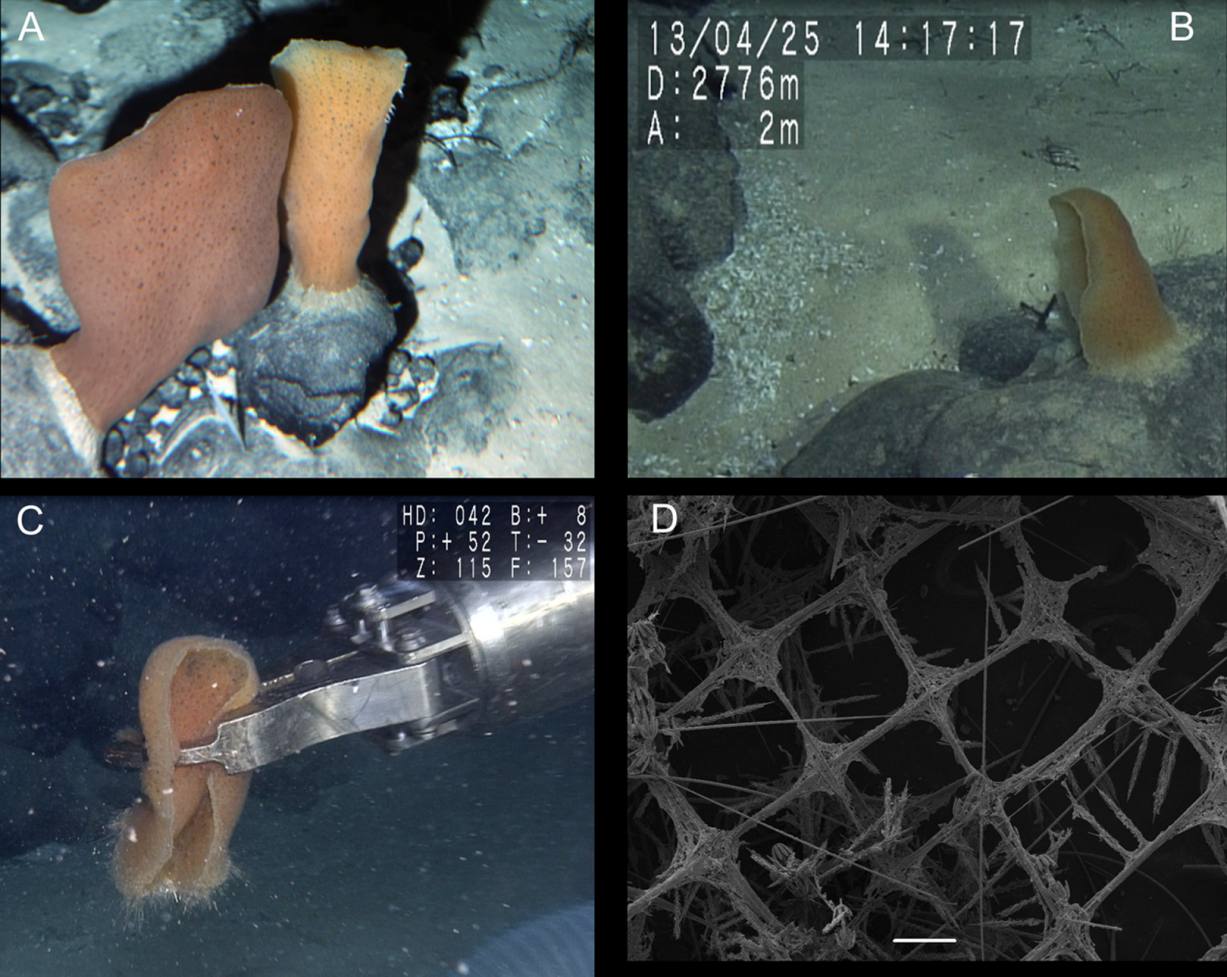
*Poliopogon amadou* specimen (MNRJ 17629). (A–C) Specimen in situ, (B and C) holotype; (D) Detail of skeleton meshes (200 μm).

**Figure 3:**
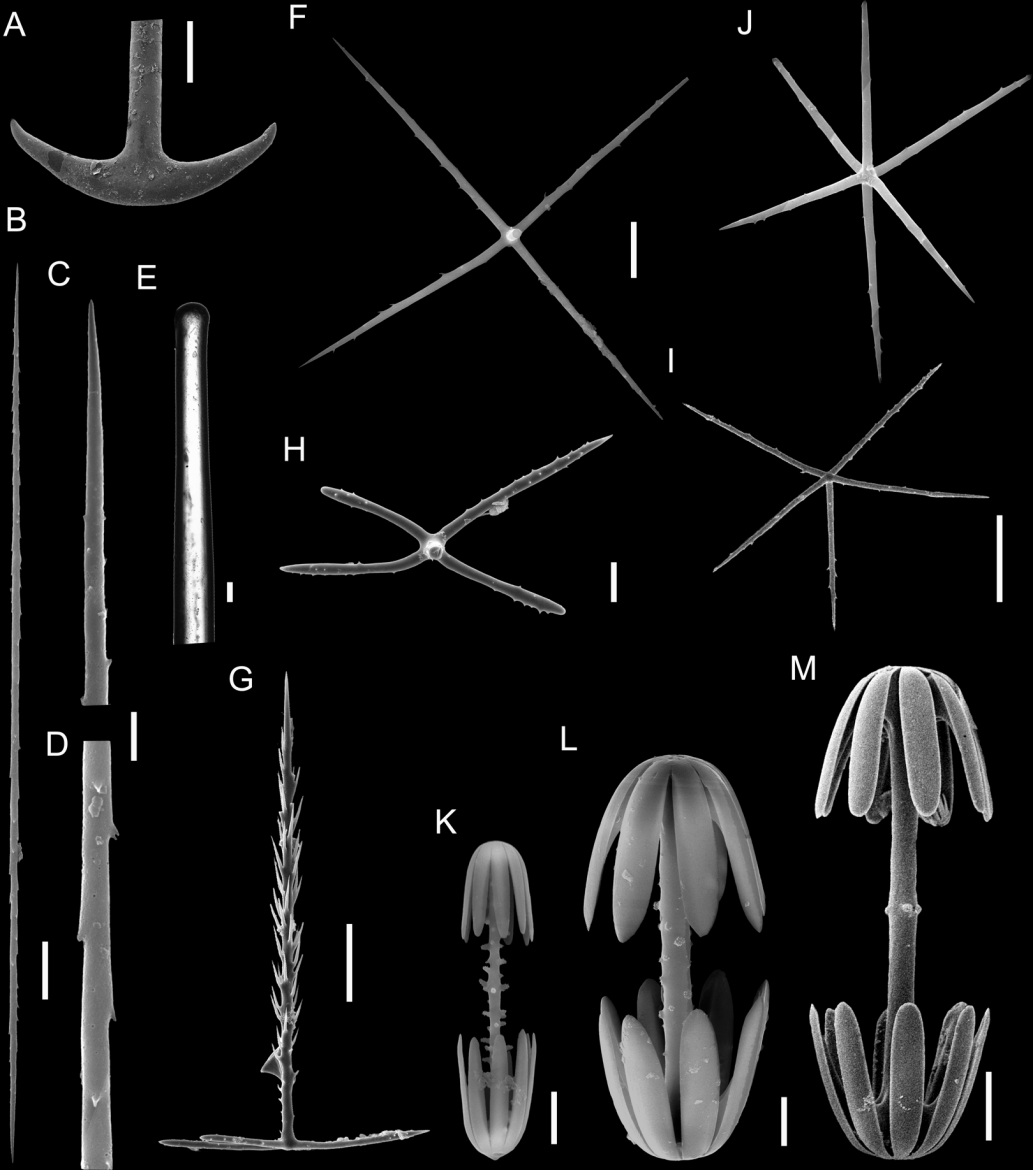
Spicules of *Poliopogon amadou*. (A) Anchorate basalium detail; (B) uncinate; (C and D) uncinates ends; (E) clavate monaxons detail; (F) Choanosomal pentactin; (G) pinular pentactin; (H) detail of pinular pentactin tangential rays; (I) microsclere pentactin; (J) microsclere hexactin; (K) micramphidisc; (L) mesamphidisc; (M) macramphidisc. Scales: (A) 100 μm; (B, C, D, F, I and L) 20 μm; (E) 2 μm; (G and J) 50 μm; (K) 10 μm; (M) 30 μm.

Examined Material

MNRJ 17629, São Paulo Ridge, Southwest Atlantic (‘Shinkai’ submersible Dive 1335—sample 6(3), Iata Piúna Expedition, 28°29′53.88″S/41°39′11.88″W), 3,060 m depth, coll. A. Augustin, 26.IV.2013.

DESCRIPTION. Lophophytous sponge, semi-funnel to fan-shaped body, following Schulze (1887) “leaf rolled up in a semi-funnel, with a concave gastral, and a convex external surface”. Specimen 173 mm long (including 12 mm long basalia), and 67 mm in diameter (adjacent to basalia), compressible and rough (fig. 2). Basalia composed of broad tufts of anchoring spicules. Brown color in situ and after preservation in ethanol.

SKELETON AND SPICULES. Quadrangular framework composed of regular and pinular pentactins, where the latter have their pinular ray piercing the sponge inwards. Basalia with two-toothed smooth anchorates, 371–543 µm wide anchors; smooth monaxons with clavate distal ends (100–229 µm diameter), and sceptres with three-toothed distal ends (1,201–2,946 × 4–10 µm). Dermalia and atrialia consisting of smooth pentactins with conical ends forming quadrangular meshes (sagittal rays, 552–2,619 × 19–29 µm; tangential rays, 368–621 × 19–29 µm); pinular pentactins with pinular rays perpendicular to the plane of the quadrangular meshes (dermal—pinular ray 310–533 × 7–10 µm; tangential rays 77–126 µm; atrial – pinular ray 252–407 × 7–10 µm, tangential rays 58–97 µm). Uncinates in two size categories: macrouncinates (795–3,589 × 4–10 µm), and mesouncinates (76–192 × 1–2 µm). Microscleres include amphidiscs in three size categories: macramphidiscs with smooth shafts (116–261 µm long, umbel 38–72 × 43–77 µm height × width), mesamphidiscs (36–68 µm long, umbel 12–19 × 9–19 µm height × width), and micramphidiscs (21–36 µm long, umbel 7–14 × 7–12 µm length × width). The latter two with spined shafts. Microhexactines (diameter 81–127 µm, ray width 3–5 µm), micropentactines (diameter 74–146 µm, ray width 2–5 µm), rarely microstauractines of similar dimensions, all with spined rays (fig. 3). Rare hexadiscs.

DISTRIBUTION AND ECOLOGY. Former records from the Azores (Thomson, 1873), and the Canary Islands (Thomson, 1877, Schulze, 1887), 2,789–4,022 m deep (Tabachnick & Menshenina, 2002). This is the first finding of the species in the South Atlantic, where it is thus far only known from the São Paulo Ridge (SW Atlantic) (present paper), at 3,060 m depth. The single specimen collected was growing on basaltic rock with other sponges likely of the same species, albeit in very low densities. No epibiontic macro- or megafauna could be seen from the images obtained (Fig. 2).

Remarks

The single specimen collected by the Iata Piuna expedition matches the original description of Thomson (1877) and redescription by Tabachnick & Menshenina (2002). The latter authors mentioned that whereas macramphidiscs are clearly separable from the smaller amphidiscs, separation of second and third categories as mesamphidiscs and micramphidiscs is not always straightforward. It is not clear to us whether Tabachnick & Menshenina (2002) were referring to every specimen as a rule, the holotype included. Regardless, in our material the three categories were clearly separable, if not entirely on the basis of dimensions, but surely when the micromorphology of these microscleres was taken into account. The rare hexadiscs seen in our specimen had previously been reported from a single specimen from the Great Meteor Bank (29°58′51.5604″N/28°29′12.6456″W, 2,480–2,550 m depth; Tabachnick & Menshenina, 2002).

Subclass Hexasterophora Schulze (1886)

Order Lyssacinosida *Zittel (1877)*

Family Euplectellidae *Gray (1867)*

Subfamily Euplectellinae Gray (1867)

Genus *Euplectella* *Owen (1841)*

Diagnosis

The body is tubular with numerous lateral oscula and usually possesses a colander-like sieve-plate. Lophophytous, attached to substratum with anchor-like basalia. Principal choanosomal spicules (large) are chiefly stauractins usually with hexactins or pentactins. The distal rays of these hexactins and pentactins are rough; the proximal rays in hexactins are always rudimentary. Additional choanosomal spicules are diactins, tauactins and rarely stauractins together with rare derivatives. The choanosomal spicules form longitudinal and circular skeleton beams. The sieve-plate, when present, contains hexactin derivatives that vary in different species. Basalia are anchor-like spicules with four or more teeth. Dermalia are hexactins. Atrialia are pentactins. Microscleres are floricomes and graphiocomes, sometimes hexasters and small sigmatocomes, rarely discohexasters, hemihexasters, hexactins and onychasters (modified from Tabachnick, 2002).

Remarks: We emended the diagnosis to include the present new species which lacks a sieve-plate. The new species fits with all the remaining aspects of the previously accepted diagnosis of *Euplectella* (cf. Tabachnick, 2002).

Considering the remaining genera of Euplectellinae, the new species described below does not fit with *Acoelocalyx* Topsent (1910) because it has hexactins, pentactins and rarely stauractins as choanosomal spicules, as well as discohexaster microscleres. It differs from *Chaunangium* Schulze (1904) by the presence, in the latter, of several distinctly separated tufts of basalia, diactins as choanosomal spicules, and discohexasters and plumicomes as microscleres. *Docosaccus* Topsent (1910) differs by the presence of diactins in the choanosoma and possession of a varied set of microscleres including hexactins, hemihexasters, hexasters, floricomes and discohexasters. It does not fit with *Holascus* Schulze (1886) because species of this genus have pinular hexactines as dermalia and atrialia, and lack floricomes. *Malacosaccus* Schulze (1886) presents choanosomal spicules which are chiefly hexactins, pinular hexactins as dermalia and atrialia, and hexaster microscleres. To the latter, hemihexasters and hexactins, discohexasters, floricomes and onychasters may sometimes be added. Finally, *Placopegma* Schulze (1896) differs by its choanosomal diactins and discohexaster microscleres, occasionally combined with plumicomes, hexasters, hexactins and discohexactins.

*Euplectella sanctipauli* sp. nov.

(Figs. 4 and 5)

**Figure 4:**
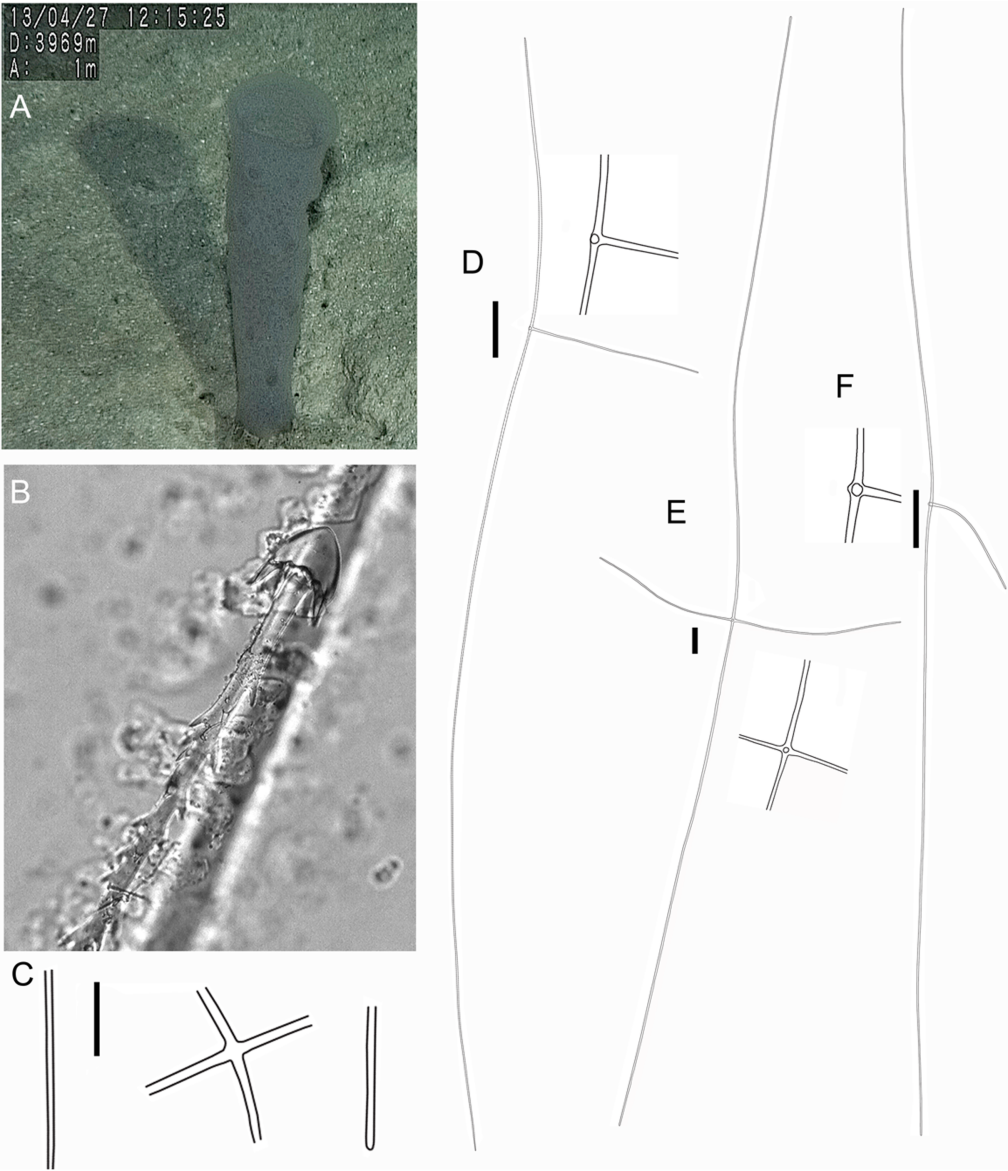
Holotype of *Euplectella sanctipauli* sp. nov. (MNRJ 17630). (A) Specimen in situ; (B–F) Megascleres: (B) Detail of anchorate basalium (μm); (C) Stauractin (100 μm); (D) Choanosomal pentactin; (E) Choanosomal hexactin; (F) Dermal/atrial pentactin with a tubercle distal ray. Scales: (B) 50 μm; (C) 100 μm; (D–F) 200 μm.

**Figure 5:**
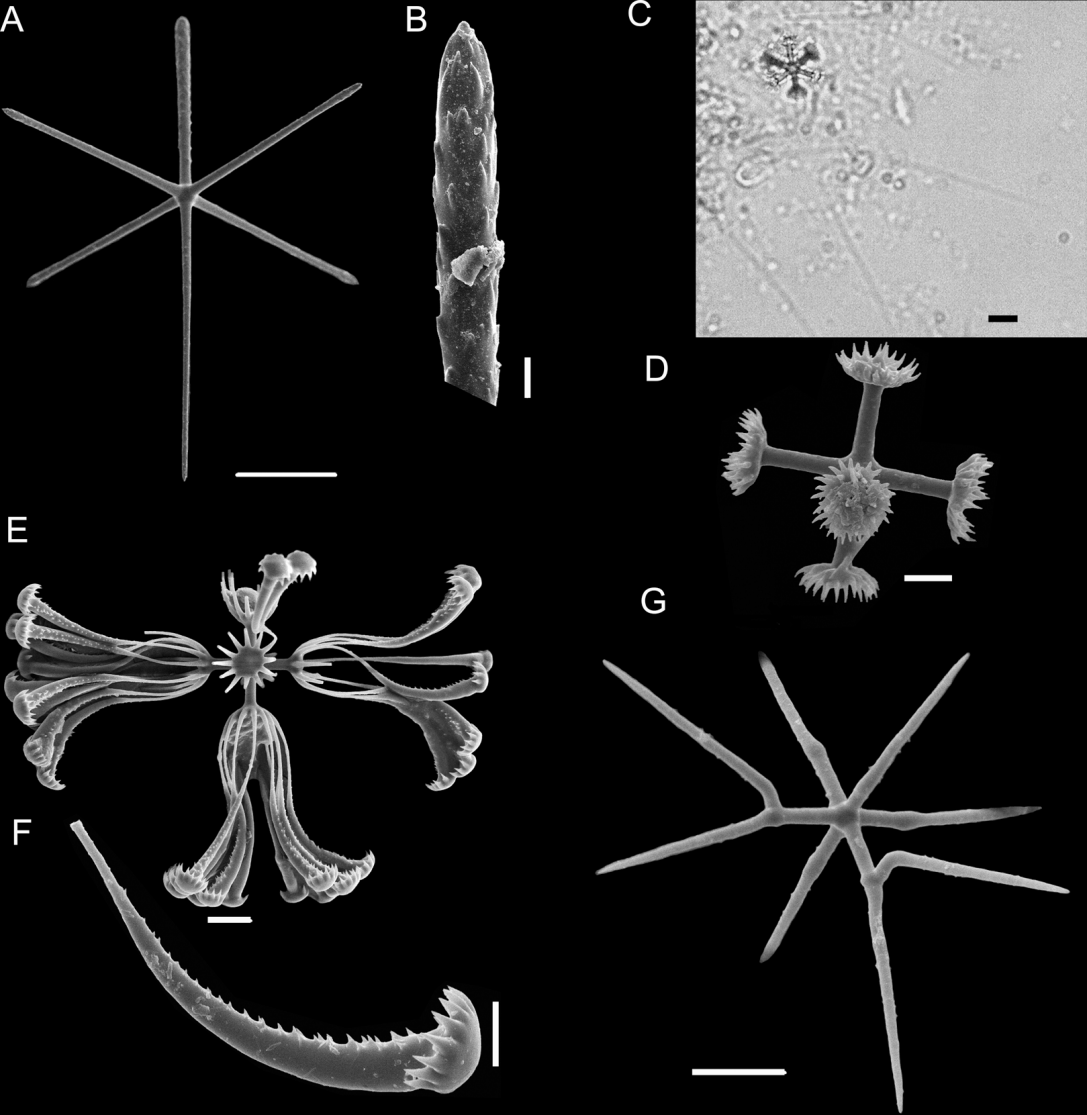
*Euplectella sanctipauli* sp. nov. spicules. (A) Dermal/atrial hexactin with sword-shaped distal ray; (B) Detail of hexactin sword-shaped distal ray; (C) Graphiocome and secondary rays; (D) Graphiocome without secondary rays; (E) Floricome; (F) Detail of apical portion of floricome secondary ray; (G) Hemioxyhexaster. Scales: (A) 100 μm; (B and C) 10 μm; (D and F) 5 μm; (E and G) 10 μm.

Type Material

Holotype. MNRJ 17630, São Paulo Ridge, Southwest Atlantic (‘Shinkai’ submersible Dive 1337—sample 5(1), Iata Piúna Expedition, 28°24′11.88″S/40°58′53.76″W), 4,061 m depth, coll. E.P. Frazão, 29.IV.2013

LSID: urn:lsid:zoobank.org:act:8688B44F-7CAC-4A5B-8EBE-07400366CA9C

Diagnosis

Only known *Euplectella* without sieve-plate. Principal skeleton composed by long, smooth stauractins, choanosomal hexactins and pentactins, dermalia/atrialia as hexactins with reduced sword-shaped distal ray with low proclined scales, or reduced as a tubercle (pentactins). Basalia as anchorate spicules with spined shaft and eight teeth on the head. Microscleres: floricomes, graphiocomes and hemioxyhexasters.

EXTERNAL MORPHOLOGY. The sponge consists of a delicate, thin-walled tube (Fig. 4) bearing a conspicuous surface reticulation, with dispersed oscula up to 4 mm in diameter, and basalia in a single tuft. Holotype (only specimen available), 137 mm long (including 34 mm long basalia/prostalia), 32 mm in maximum diameter.

SKELETON AND SPICULES (Figs. 4 and 5). Principalia mostly stauractins with rays 2.4–5.9 mm long, 12–17 µm in diameter (*n* = 4), and elongated or rounded ends. Choanosomal spicules long, smooth hexactins (1.1–5.1 mm long rays (*n* = 10), elongated or rounded ends) and pentactins (3.8 – 7.6 mm long rays (*n* = 5), rounded ends). Dermalia and atrialia hexactins with a reduced and sword-shaped distal ray with low proclined scales (0.2–0.3 mm long), and tangential rays with elongated and rounded ends (0.3–0.4 mm long); pentactins with distal ray reduced as a tubercle, and smooth tangential rays (1.9–2.1 mm long; *n* = 2). Basalia composed of anchorate spicules with spiny shafts, and anchors with eight teeth. The largest spicule (broken) was 8.5 mm long.

Microscleres are floricomes, graphiocomes and hemioxyhexasters. Floricomes with spined rays, and eight teeth on secondary rays, this last in number of 10–12 in each primary ray; diameter 110–130 µm, primary rosette 10–17 µm diameter. Graphiocomes observed with secondary rays always broken (68–85 µm long), primary rosette 10–14 µm diameter. Hemioxyhexasters (95–118 µm in diameter, *n* = 4) with two or three unbranched rays (45–50 µm in diameter); branched rays divide 7–10 µm from the origin and have secondary rays of length 10–20 µm (*n* = 4). (Fig. 5F).

DISTRIBUTION AND ECOLOGY. Known only from its type locality, São Paulo Ridge (Southwest Atlantic; 4,061 m depth). The single specimen was collected from soft sediment, apparently the sole megafauna in several meters of terrain, and seemingly devoid of any macro-epibionts.

ETYMOLOGY. The specific epithet is used as a noun in apposition, derived from the species having been found at the São Paulo Ridge (deep SW Atlantic).

Remarks

*Euplectella* comprises 18 species around the world (Reiswig & Kelly, 2018), classified by Tabachnick & Collins (2008) into four main groups recognizable on the basis of the architecture of their principal skeleton: (1) mainly stauractins, (2) mainly hexactins with a reduced proximal ray and some stauractins, (3) pentactins and some hexactins and (4) stauractins, hexactins, hexactins with reduced proximal ray, hexactins with two reduced rays, tauactins and diactins. *Euplectella sanctipauli* sp. nov. should be included in the first group, in addition to *E. aspergillum* Owen (1841), *E. curvistellata* *Ijima (1901)*, *E. gibbsa* Tabachnick & Collins (2008), *E. marshalli* *Ijima (1895)*, *E. oweni* *Herklots & Marshall (1868)*, *E. paratetractina* *Tabachnick, Janussen & Menschenina (2008)* and *E. simplex* Schulze (1896). The present species appears most similar to *E. aspergillum*, *E. gibbsa* and *E. simplex*, but differs from all of them by its possession of anchorate basalia with eight teeth, choanosomal hexactins and pentactins, and dermalia and atrialia including hexactins with a reduced and sword-shaped distal ray, as well as additional pentactins with distal ray reduced as a tubercle. In addition, *E. aspergillum* differs in having anchorate basalia with six teeth, and diactins; *E. gibbsa* differs in having anchorate basalia with four teeth, choanosomal tauactins and diactins; and *E. simplex* differs by its triactins and diactins. To date, this is the only *Euplectella* species without a sieve-plate, which clearly establishes *E*. *sanctipauli* sp. nov. as a new species.

Subfamily Bolosominae Tabachnick (2002)

Genus *Bolosoma* *Ijima (1904)*

Diagnosis

Body is fungiform, pedunculate, basiphytous with a more-or-less everted atrial cavity. Choanosomal spicules predominantly diactins, rarely hexactins and pentactins too. The spicules of the peduncle are diactins fused into a rigid skeleton by synapticular junctions. Dermalia and atrialia are hexactins, rarely pentactins. Microscleres are anchorate and toothed discohexasters, discasters, hemidiscohexasters, discohexactins, and derivatives of the latter to amphidiscs (Tabachnick, 2002 modified by Reiswig & Kelly (2011)).

*Bolosoma perezi* sp. nov.

(Figs. 6 and 7)

**Figure 6:**
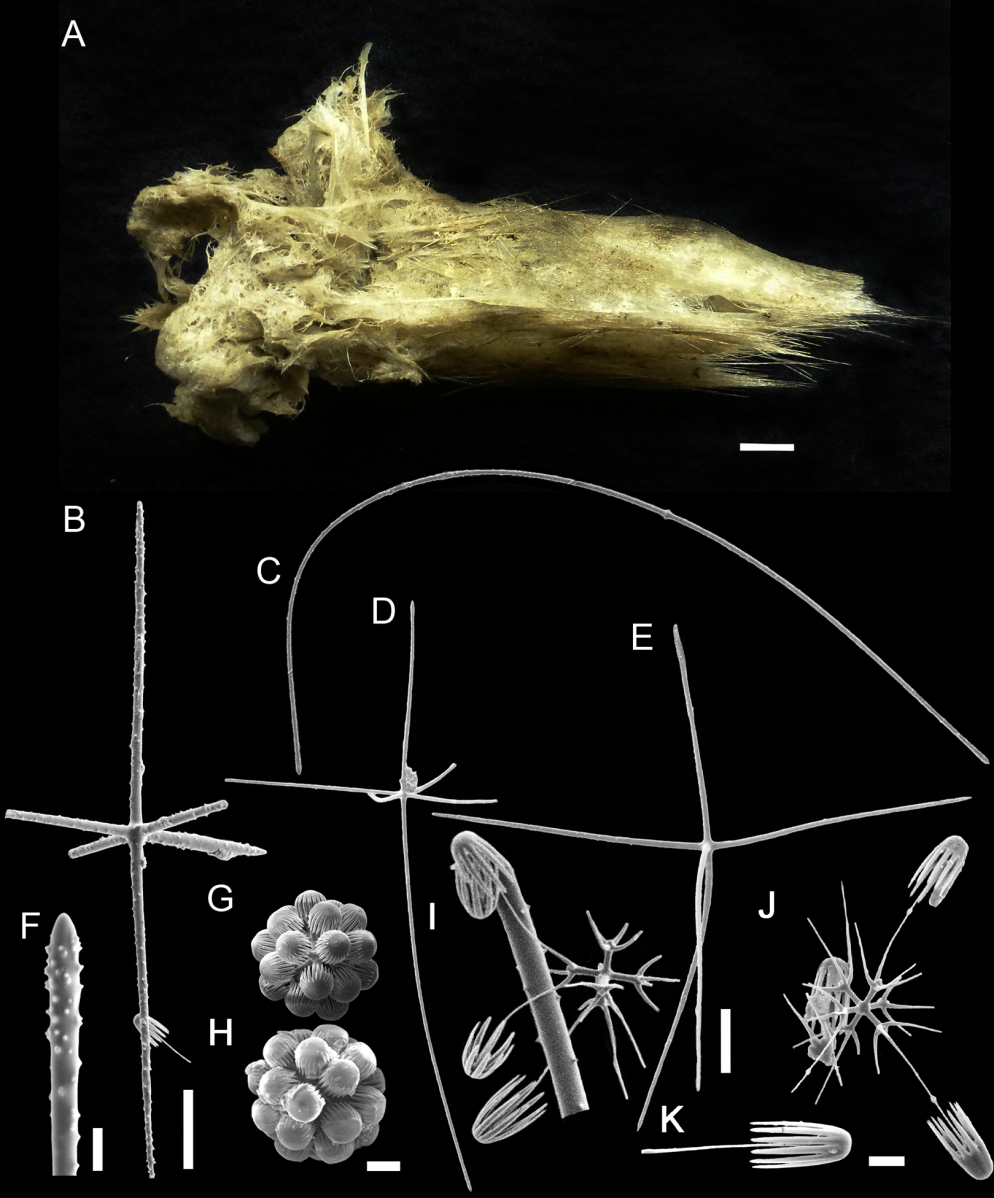
*Bolosoma perezi* sp. nov. holotype (MNRJ 21955). (A) Specimen; (B, D and E) hexactins; (C) Choanosomal diactin; (F) detail of hexactin’s distal end of ray; (G and H) discohexasters; (I and J) codonhexasters; (K) anchorate end of a codonhexaster. Scales: (A) 10 mm; (B) 50 μm; (F, I, J and K) 10 μm; (C–E) 100 μm; (G and H) 20 μm.

**Figure 7:**
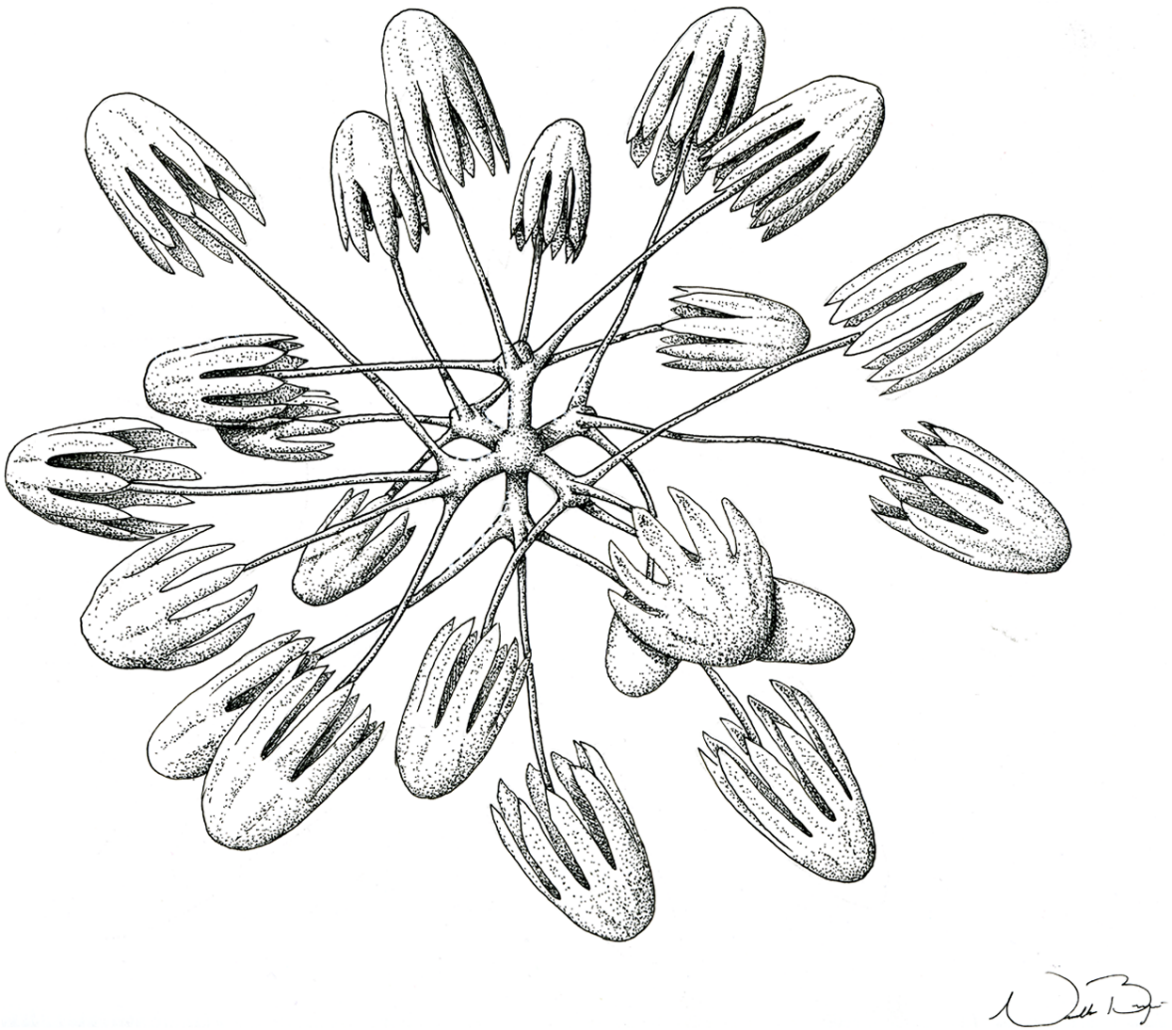
Schematic illustration of *Bolosoma perezi* sp. nov. codonhexaster (Illustration by Nicholas Bezio).

Type Material

Holotype. MNRJ 21955, Rio Grande Rise, Southwest Atlantic (PROERG Expedition, ERG 15—L2; 31°7′3.36″S/34°1′48.72″W), 1,022–1,013 m depth, 07.VII.2011.

LSID: urn:lsid:zoobank.org:act:CAD5EDB9-DB39-4684-A160-95EA2FA21210

Diagnosis

*Bolosoma perezi* sp. nov. is the only *Bolosoma* with discohexasters and codonhexasters microscleres.

EXTERNAL MORPHOLOGY. Sponge fragment, cushion-shaped and compressible, 162 mm in length × 78 mm in width × 16–27 mm in height; fragment apparently without a peduncle, color light gray in ethanol (fig. 6).

SKELETON AND SPICULES. A few long cemented diactins (40–80 μm diameter and over 900 μm long), which we assume are fragments of a peduncle. Choanosomal diactins with rough ends and rudiments of tangential rays (1,275–3,125 × 5–8 μm). Dermal and atrial hexactins and rare atrial pentactins with rough ends and sometimes random spines along the ray (270‒830 × 5‒8 μm). Microscleres are ball-like discohexasters (70‒80 μm diameter), its derivates (stauractins, 42–50 μm diameter), and codonhexasters (155–175 μm diameter, primary rosettes 27–43 μm diameter). The last one with six primary rays and secondary rays in number of four; discs with nine teeth (fig. 7).

DISTRIBUTION AND ECOLOGY. Known only from its type locality in the Rio Grande Rise (Southwest Atlantic), 1,022–1,013 m depth. This is the first record of *Bolosoma* for the entire Atlantic Ocean.

ETYMOLOGY. The specific epithet honors Prof. Dr. Angel Perez (UNIVALI, Brazil) for granting us access to Rio Grande Rise materials, and for our long-standing collaboration in the study of South Atlantic deep-sea ecosystems.

Remarks

*Bolosoma* comprises eight species (see comparative Table 1), up to now recorded only from the Pacific Ocean. The new species presents a unique set of spicules, indicating its distinctiveness from the remaining species in the genus. None of its congeners present the combination of delicate codonhexasters and “ball-like” discohexasters, which confers *B. perezi* sp. nov. obvious status as a new species. This is the first record of *Bolosoma* for the Atlantic Ocean.

**Table 1:** Comparative data for habit, anatomy, spicules and occurrence for *Bolosoma* spp. Data for new species presently generated. Data for previously known species gathered from the literature. All morphometric data are in micrometers (µm). n.o., not observed.

	Body	Basalia	Choanosoma/Dermalia/Atrialia	Microscleres	Distribution/Depth (m)
*B. perezi* sp. nov. (holotype, MNRJ 21955)	Fragment of sponge, massive without a regular shape	Diactines, 40–80 diam. Occasionally free diactines, 15–20 diam.	Diactines, 1,710–2,370 × 5–8 Hexactines, 270–830 × 5–8 Rare pentactines	Discoxactines, absent Discohexasters, 70–80 Discostauractines, 42–50 Codonhexasters, 155–175 (primary rosettes 27–43 diam.)	Rio Grande Rise, South Atlantic Ocean/936
*B. biocalum* *Tabachnick & Lévi (2004)* (orig. descr.)	Two fragments	Diactines, 19–23 diam.	Diactines, 900–5,000 × 6–15 Hexactines (uncommon pentactines), distal actin 68–258 µm long, tangential actin 106–274 µm long, proximal actin 258–775 µm long; all actines 6–11 diam.	Discohexactines, absent Discohexasters, 61–108 (primary rosettes, 7–14 diam.)	New Caledonia/1,395–1,620
*B. cavum* *Ijima (1927)* (orig. descr.)	Vase–like shape standing on a compact basal disc	Diactines, up to 50 µm thick, up to 7,000‒8,000 long Occasionally hexactines or pentactines	Hexactines, short ray 55–88 long, 7.5–11 thick near base; paratangential rays 180–266 long	Discohexactines, absent Spherical holodiscohexasters, 113–133 diam. Hexactinoid microdiscohexaster (occasionally hemidisco.), 23–38 (axial lenght), in one case 50. Hexactinoid codonhexaster, 152–180 diam.	Sulawesi Sea, Makassar Strait/918–1,613
*B. charcoti* Tabachnick & Lévi (2004) (orig. descr.)	Holotype composed by a fragment of the apical region, paratype fragmented and attached to a peduncle	Not reported	Diactines, 14–53 diam. Tauactins, uncommon Hexactines (dermal or atrial), distal actines 30–167 long; tangential actines 106–380 long; proximal actines, 460–532 long Atrial hexactines, distal actines 30–547 long; tangential actines 61–365; proximal actines, 30–106; all with 7–9 diam.	Toothed discohexactins, 32–86 diam. Discohexactines with anchors (less common), 68–144 diam.	New Caledonia/1,300–1,475
*B. cyanae* Tabachnick & Lévi (2004) (orig. descr.)	Pedunculated champignon	Diactines, 8–46 diam. Hexactines, distal actine with 400–600; tangential actine 500–1,100; proximal actine 400–1,400; all actines 30–53 diam.	Diactines, 1,200–3,800 × 8–15 Hexactines (dermal region), distal actine 65–250 long; tangential actines 95–470; proximal actine 220–710 long; 10–17 diam. Hexactines (atrial region), distal actines, 110–680 long.; tangential actines 190–700; proximal actine 40–210 long; 10–17 diam.	Microdiscohexactines, 20–47 diam. Macrodiscohexasters, 65–195 diam. (primary rosette, 10–23 diam. Microdiscohexasters, 72–145 diam. Rare hemidiscohexasters, same diam. of microdiscohexasters.	Loyauté Basin (New Caledonia)/2,697–2,380
*B. meridionale* Tabachnick & Lévi (2004) (orig. descr.)	Pedunculated sponge with apical part in funnel form	Diactines, 8–61 diam.	Diactines, 1,300–1,900 × 7–9 Hexactines (dermal region), distal actines 53–114 long, tangential actines 99–205, proximal actine 296–486 µm; 10–17 diam. Hexactines (atrial region), distal actines 68–433; tangential actines 137–319; proximal actines 46–106; 10–17 diam.	Discohexactines, 50–72 diam. Hemidiscohexasters, 47–79 diam. (primary rosette, 7–9 diam.) Discohexactine derivates, 50–72 diam.	S–E New Caledonia/995–1,010
*B. musorstomum* Tabachnick & Lévi (2004) (orig. descr.)	A fragment of peduncle with an apical part	Diactines, smooth, cemented	Diactines, 1,400–1,900 × 6–8 Hexactines (dermal region), distal actine 30–190 long, tangential actines 38–410, proximal actine 46–471; 19–22 diam. Hexactines (atrial region), distal actines 68–411; tangential actines 152–274; proximal actine 30–137; 19–22 diam.	Discohexactines, 15–36 diam. Discostauractines, discotauactines and discodiactines (“amphidiscs”)	Wallis Island, New Caledonia/775–730
*B. paradictyum* (*Ijima, 1903*) (orig. descr.)	Fungus–like body with everted atrial cavity	Diactines fused into a rigid skeleton by synapticulars.	Diactines, 2,000–8,000 × 15 Hexactines (dermal region), distal ray 45–100 long; tangential rays 150–300 long; 8–9 diam. at base Hexactines (dermal region), tangential rays 200–260 long; proximal ray 600–800 long; 8–9 diam. at base	Microdiscohexactines, 30–60 diam. Toothed microhemidiscohexasters, 30–100 diam. Toothed macrodiscohexasters, 100–240 diam. Toothed microdiscohexasters, 30–100 diam. Anchorate macrodiscohexasters, 132 diam. Anchorate macrodiscohexactines, 110–176 diam.	Sagami Bay, (Japan)/501–572
*B. volsmarum* Tabachnick & Lévi, 2004 (orig. descr.)	Fragment of peduncle, which carries an apical, incomplete part.	Diactines, smooth, cemented	Diactines, 1500–3200 x 8–15 µm Hexactines (dermal region), distal actines 91–228 µm long; tangential actines 182–418 µm; proximal actines 365–874 µm; 15–19 µm diam. Hexactines (atrial region), distal actines 593–737 µm; tangential actines 236–692 µm; proximal actine 38–160 µm; 15–19 µm diam.	Discohexactines, 32–65 diam. Discohexasters, 54–126 diam. (primary rosette, 11–22 diam.)	S–E New Caledonia / 825–850

Genus *Advhena* gen. nov.

LSID: urn:lsid:zoobank.org:act:C9A45855-9AA8-4088-8665-9830A8CC59AB

**Type species:** *Advhena magnifica* gen. et sp. nov. (described below)

Diagnosis

Bolosominae with a globular body slightly flattened, with big lateral opening and long stalk (at least four times the body size). Choanosomal spicules are diactins. Dermalia and atrialia are hexactins and pentactins. Microscleres are discohexasters, codonhexasters, calycodiscohexasters and graphiocomes.

Etymology

Feminine gender. Modified from the latin, ‘Advena’, meaning stranger, foreigner, alien, newcomer, guest, in reference to the sponge shape, which calls to mind aliens from various movies. An ‘h’ was added in ‘Advena’ (*Advhena*) to distinguish the name from that of the helicarionid gastropod *Advena* Gude, 1913, in order to make sure there will be no overlapping with valid names.

Remarks

The new specimen cannot be accommodated in any of the currently accepted genera in the subfamily (Table 2). It is clearly a long stalked Bolosominae individuum, with diactins as the main spicules, plus hexactins and pentactins. It is distinguished from other Bolosominae by the set of microscleres present, namely discasters, discohexasters, codonhexasters and derivatives, calycocomes, and graphiocomes, which render it unique among bolosomines, and justifies the proposal of a new genus.

**Table 2:** Comparative data for habit, anatomy, spicules and occurrence for Bolosominae genera. Data for new genus presently generated. Data for previously known genera gathered from the literature. n.o., not observed.

	Body	Peduncle/Basalia	Choanosoma/Dermalia/Atrialia	Microscleres	Graphiocomes/Floricomes/Plumicomes
*Advhena* gen. et sp. nov.	Champignon–like inverted with lateral openings concentrated on one side	Long peduncle Fused diactins Short hexactines	Diactins Hexactins Rare pentactins	Discohexasters (type species with two types of) Codonhexasters Calycodiscohexasters	Graphiocomes
*Amphidiscella* *Tabachnick & Lévi (1997)*	Cup-like	Rigid tubular peduncle Fused diactins	Diactins Hexactins Paratetractins	Amphidiscs Staurodiscs Hexadiscs Discohexasters Hemidiscohexasters Sigmatocomes (sometimes)	Floricomes
*Amphoreus* Reiswig & Kelly (2018)	Thick-walled, funnel-shape	Short thick peduncle Fused diactins	Diactins Sword hexactins (dermalia) Hexactins (atrialia)	Discohexasters Stellate-floricoid discohexasters Hexasters Oxystauraster	n.o.
*Bolosoma* Ijima (1904)	Fungus–like body with a more-or-less everted atrial cavity	Pedunculate Fused diactins	Diactins Hexactins Pentactins	Discohexactines Hemidiscohexasters Discohexasters and derivates	n.o.
*Caulocalyx* Schulze (1886)	Cup-like	Pedunculate Fused diactins	Diactins Pentactins (dermalia) Hexactins (atrialia)	Discohexasters	Plumicomes
*Hyalostylus* Schulze (1886)	Bell-like or discoidal upper part and vast atrial cavity	Long peduncle Fused diactins	Diactins Hexactins	Sigmatomes Discohexasters (spherical and stellate) Hexasters	Floricomes
*Neocaledoniella* Tabachnick & Lévi (2004)	Fungus-like with completely everted atrial cavity	Long tubular peduncle Fused diactins	Diactins Pinular hexactins	Calycodiscohexasters (calycocomes)	Graphiocomes
*Rhizophyta* Shen et al. (2019)	Fungus-like with completely everted, laterally directed atrial cavity	Long, root-like outgrowths peduncle Fused diactins Pentactins	Diactins Pinular hexactins Rare pentactins	Stellate discohexasters	n.o.
*Saccocalyx* *Schulze (1896)*	Cup-like with large atrial cavity and thin walls	Long tubular peduncle Fused diactins	Diactins Hexactins Pinular hexactins	Spirodiscohexasters Codonhexasters Drepanocomes Acanthose microxyhexactins	Plumicomes
*Trachycaulus* Schulze (1886)	Spherical body	Long, rigid peduncle Fused diactins	Diactins Pinular hexactins	Drepanocomes	Graphiocomes
*Trychella* Reiswig & Kelly (2018)	Cowbell-shape	Short hard peduncle	Diactins Hexactins Sword Hexactins (dermalia)	Amphidiscs Tetradiscs and derivates Dicasters Discohexasters Microxy diactins	Graphiocomes with helicoid terminal rays (spiroxyhexasters)
*Vityaziella* Tabachnick & Lévi (1997)	Cup-like	Long peduncle Fused diactins	Diactins Hexactins	Amphidiscs	Graphiocomes

*Advhena magnifica* sp. nov.

(Figs. 8–10)

**Figure 8:**
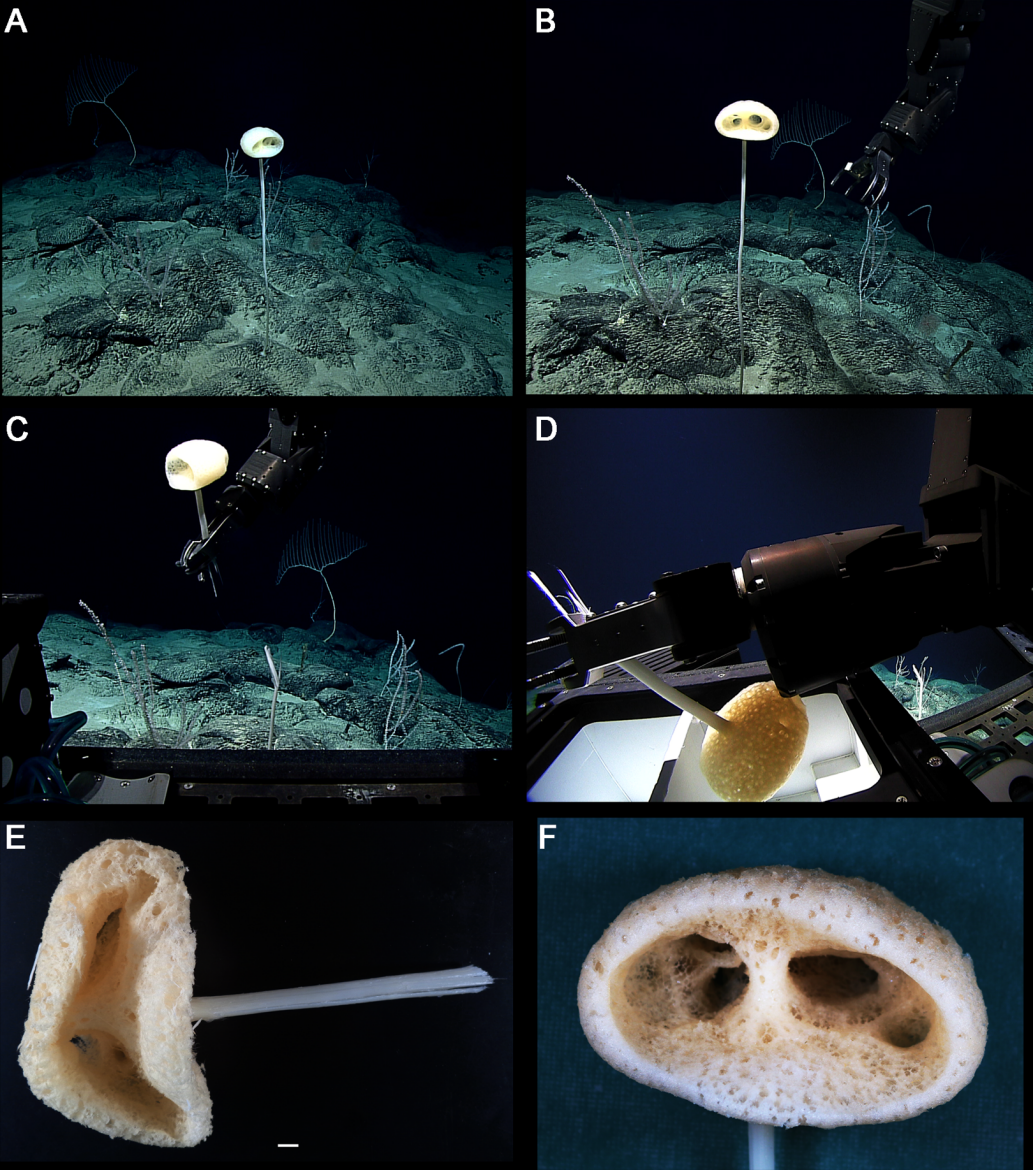
*Advhena magnifica* gen. et sp. nov. holotype (USNM 1424107). (A‒D) specimen in situ; (E and F) details of specimen in ethanol (1 cm).

**Figure 9:**
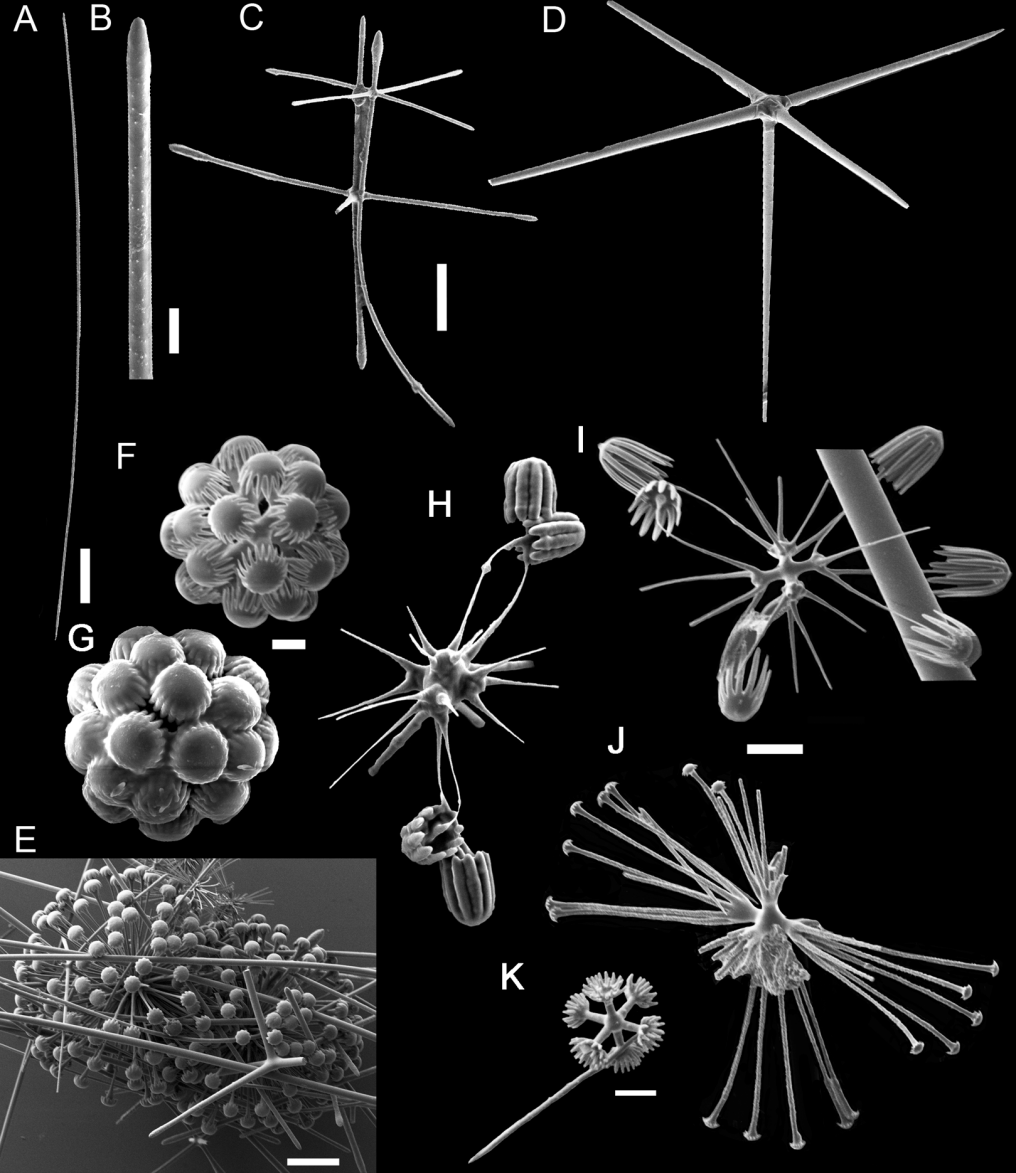
*Advhena magnifica* sp. nov. (A) diactin; (B) detail of hexactin extremity; (C and D) pentactines; (E) discasters (discohexaster); (F and G) discohexaster; (H and I) codonhexasters (J) calydiscohexasters (partially broken); (K) graphiocome (primary rays with one of the secondary rays). Scales: (A) 250 μm; (B, H, I and J) 20 μm; (C–E) 100 μm; (F, G, J and K) 10 μm.

**Figure 10:**
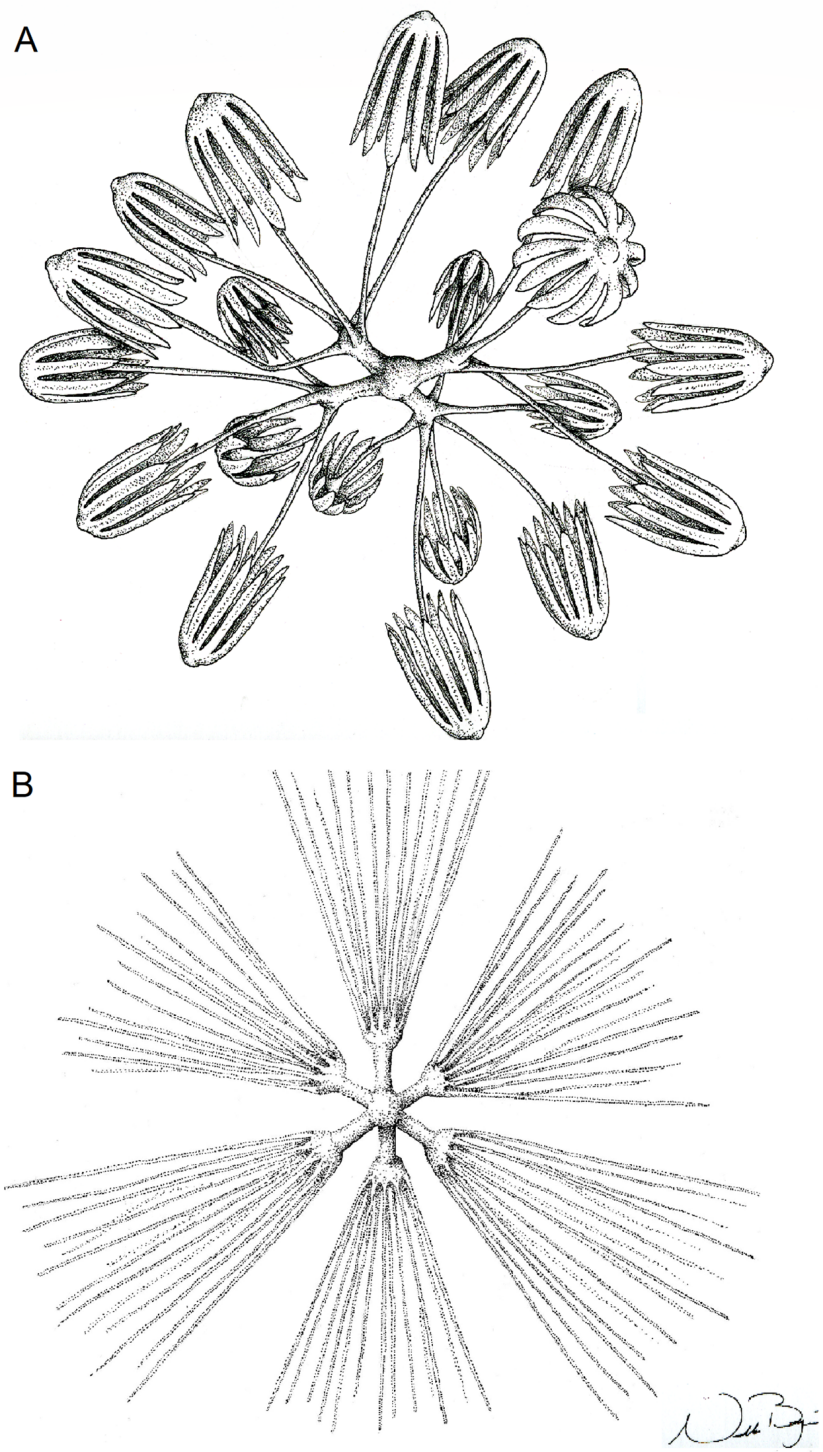
Illustration of *Advhena magnifica* gen. et sp. nov. microscleres. (A) Codonstauraster; (B) graphiocome (Illustrations by Nicholas Bezio).

Type Material

Holotype. USNM 1424107. Pigafetta Guyot, E of the Marianas’ Trench, Pacific Ocean (Deepwater Exploration of the Marianas, ‘Deep Discovery’ ROV; Cruise EX1605L1; 15°56′31.3836″N/148°36′53.3556″E), coll. R/V ‘*Okeanos Explorer ’*, 2,028 m depth, 04.V.2016.

EZID: http://n2t.net/ark:/65665/31f4b11ee-5685-4b5b-befb-58efb94b3c6c

LSID: urn:lsid:zoobank.org:act:52622D46-619E-432B-9D80-77795C5FD8A7

Diagnosis

*Advhena magnifica* sp. nov. is the only representative of Bolosominae with microscleres as discasters (480–570 μm diam.), discohexasters (55–60 μm diam.), codonstaurasters (103–160 μm diam.), discohexasters with calycocomes (138–255 μm diam.) and graphiocomes (150 μm (*N* = 1); 20–33 μm primary rays’ diam.).

EXTERNAL MORPHOLOGY. Pedunculate sponge characterized by mushroom-like shape (136 mm in diameter and 75 mm thick) with lateral openings concentrated on one side of the body, each one with 50 and 18 mm in diameter, and peduncle longer than 154 mm in length (broken) and 16 mm in diameter (at least four times the body size).

SKELETON AND SPICULES. Peduncle with cemented diactins (1,310–2,825 × 20–60 μm) and a few short hexactins and pentactins with distal ray reduced (195–250 × 2–5 μm). Choanosomal diactins with rough ends and sometimes rudiments of actins in the middle region; 1,075–2,575 × 15–18 μm. Dermal and atrial hexactins smooth with rough ends and shorter distal ray; 300–789 × 10–18 μm, and distal ray 80–110 × 12–25 μm; rare pentactins as dermalia/atrialia (450–680 × 15–18 μm). Microscleres discasters (480–570 μm diameter); discohexasters (50–75 μm diameter); codonstaurasters (103–160 μm diameter) usually with four to six central axis and five to seven secondary rays (sometimes bent over), discs with eleven teeth; calycocomes (138–255 μm diameter), and graphiocomes (150 μm (*N* = 1); 20–33 μm primary rays’ diameter) with six central rays and straight and short secondary rays (maximum found 65 μm length).

DISTRIBUTION AND ECOLOGY. Known from its type locality in the Pigafetta Guyot, east of the Mariana Trench (Pacific Ocean), 2028 m depth. Some video footage of likely *Advhena* specimens was obtained by NOAA ‘Okeanos’ expedition a year later (25 July 2017) at a locality rich in sponge diversity, dubbed the “Forest of the Weird”, as part of the Laulima O Ka Moana: Exploring Deep Monument Waters Around Johnston Atoll expedition (EX1706; on https://oceanexplorer.noaa.gov/okeanos/explorations/ex1706/dailyupdates/media/video/dive11-forest/forest.html).

ETYMOLOGY. The specific epithet is used as a noun in apposition, and refers to the species’ magnificent, beautiful appearance.

Remarks

Considering all genera of Bolosominae (see table 2), *Advhena* gen. nov. appears most similar to *Neocaledoniella* Tabachnick & Lévi (2004), the only other bolosomine with calycocomes and graphiocomes. However, the new species has discaster, discohexaster and codonstauraster microscleres. In addition, *Neocaledoniella* presents heavily spined pinular hexactins, missing here. Thus, we propose a new monospecific genus within Bolosominae to include *Advhena magnifica* gen. et sp. nov.

Identification key to genera of Bolosominae (modified from Tabachnick, 2002):

(1) Graphiocomes present …………… 2

Graphiocomes absent …………… 6

(2) Microscleres with discoidal outer ends present …………… 3

Microscleres with discoidal outer ends absent …………… *Trachycaulus*

(3) Microscleres with discoidal outer ends are calycocomes …………… *Neocaledoniella*

Microscleres with discoidal outer ends not exclusively calycocomes …………… 4

(4) Amphidiscs present …………… 5

Amphidiscs absent …………… *Advhena*

(5) Microscleres with discoidal outer ends are exclusively amphidiscs …………… *Vitiaziella*

Other type of microscleres with discoidal outer ends besides amphidiscs …………… *Trychella*

(6) Plumicomes present …………… 7

Plumicomes absent …………… 8

(7) Only microscleres with discoidal secondary rays present besides plumicomes …………… *Caulocalyx*

Other microscleres besides plumicomes and that with discoidal outer ends present: with sigmoidal and floricoidal secondary rays …………… *Saccocalyx*

(8) Stellate discohexasters present …………… 9

Stellate discohexasters absent …………… *Bolosoma*

(9) Only stellate discohexasters as microscleres …………… *Rhizophyta*

Microscleres various …………… *Amphoreus*

## General Discussion

With our observations, South Atlantic hexactinellid diversity still only amounts to 34 species. Most of these observations are from the continental slope in the SW (20 spp) and SE Atlantic (three spp), areas much more easily accessible due to the relatively small navigational distances involved. The present study constitutes a ~27% increase in the knowledge of South Atlantic deep-sea hexactinellids not associated with continental margins (three new records, raising the total to 14 species). The area comprised by abyssal plains, the mid-Atlantic ridge, Rio Grande Rise, São Paulo Plateau, and many additional important geomorphologic features not associated to continental margins, is an enormous part of the South Atlantic (~26.46 km^2^ × 10^6^; Watling et al., 2013), but limited observations hinder understanding of hexactinellid and other biodiversity. This is obviously a major frontier for deep-sea exploration. The opportunity brought up by Brazil’s research obligations, in exchange for the permission granted by the International Seabed Authority (International Seabed Authority, 2015) for the exploration of mineral crusts at the Rio Grande Rise, is a considerable opportunity to pursue an exponential increase in the knowledge of deep-sea benthic biodiversity in this large area of the SW Atlantic.

The present study brought together materials obtained through dredging and manned-submersible (“Shinkai 6500”) collections, between 3,060 and 4,061 m depth, important tools for building a comprehensive understanding of deep-sea communities in the Rio Grande Rise. At present, only the tip of the iceberg has been touched upon. As dredging has been conducted with geological priorities in mind, biological samples are few and mostly severely damaged. On the other hand, samples obtained by the “Shinkai 6500” are of good quality by nature. Although very few in number, those samples, together with records of environmental parameters, can greatly improve understanding of deep-sea biodiversity, habitats and ecosystems. New collections should be planned with the use of ROVs, essential to establish the true dimensions of the recently described *Sarostegia* *Topsent (1904)* sponge garden, for example (Hajdu et al., 2017), as well as to determine whether other potentially vulnerable marine habitats occur in the Rio Grande Rise.

*Poliopogon amadou* is reported here for the first time from the South Atlantic. Xavier, Tojeira & Van Soest (2015) reported upon large aggregations of this species in the NE Atlantic (Meteor Bank). It is not clear to us how abundant the species is in the São Paulo Plateau, something worth investigating further, as part of the expected commitment to the study of deep-sea communities in the neighboring Rio Grande Rise over the next decades.

A new species of *Bolosoma* is described here, raising to nine the number of known species in this genus, and expanding the genus’ known distribution to the Atlantic Ocean. A better assessment of deep-sea sponge biodiversity in the Atlantic and the Pacific will only be possible with additional efforts, both in enabling new expeditions, but also in granting support to the necessary work on proper identifications of collections already available. A considerable number of deep-sea sponge specimens is deposited in scientific collections around the world, calling for taxonomists to work on them and furnish not only inventory data, but also reveal surprising animals, whose integrative study can help us build a sharper Porifera Tree of Life.

Finally, a new genus in the Pacific Ocean is proposed here within Bolosominae, *Advhena* gen. nov. The last decade has seen a significant increase in recognition of higher taxa within this subfamily, with four new genera proposed, as well as eleven new species. Including *Advhena* gen. nov., all of the recently recognized bolosomine diversity has been reported from the Pacific (*Amphoreus* Reiswig & Kelly, 2018, *Rhizophyta* Shen et al., 2019 and *Trychella* Reiswig & Kelly, 2018), further highlighting the richness of this ocean basin, which is also apparent in the number of *Bolosoma* spp. described thus far (cf. above). Although Bolosominae was found to be non-monophyletic (Dohrmann et al., 2017), the new genus fits best within it taxonomically until a detailed integrative revision of Euplectellidae and its subfamilies is completed.

## Additional Information and Declarations

### Data Availability

The following information was supplied regarding data availability: All generated data is available in the article (Results, Tables 1 and 2) and all the specimens are deposited in the Porifera collection of the Museu Nacional/UFRJ (MNRJ 17629, 17630 and 21955) and the Smithsonian National Museum of Natural History (USNM 1424107, accession number: 2078414).

### New Species Registration

The following information was supplied regarding the registration of a newly described species: Publication LSID: urn:lsid:zoobank.org:pub:19DC8DB7-AE38-4CBD-8DBE-F90799979352. Euplectella sanctipauli sp. nov. LSID: urn:lsid:zoobank.org:act:8688B44F-7CAC-4A5B-8EBE-07400366CA9C. Bolosoma perezi sp. nov. LSID: urn:lsid:zoobank.org:act:CAD5EDB9-DB39-4684-A160-95EA2FA21210. Advhena LSID: urn:lsid:zoobank.org:act:C9A45855-9AA8-4088-8665-9830A8CC59AB. Advhena magnifica sp. nov. LSID: urn:lsid:zoobank.org:act:52622D46-619E-432B-9D80-77795C5FD8A7.
